# A Nomogram Prediction Model for Internal Hernia Using Clinical Parameters and Non-Enhanced Computed Tomography Imaging

**DOI:** 10.1007/s11605-022-05429-3

**Published:** 2022-12-12

**Authors:** Yunlong Li, Zhen Tian, Chengcong Liu, Shikuan Li, Weiqun Bi, Qinglian Ji

**Affiliations:** 1 Department of Emergency General Surgery, The Affiliated Hospital of Qingdao University, Qingdao, 266003 Shandong China; 2 Department of Anorectal Center, Qilu Hospital (Qingdao), Cheeloo College of Medicine, Shandong University, Qingdao, Shandong China; 3 Department of Gastrointestinal Surgery, Qingdao Central Hospital, Qingdao, Shandong China; 4 Department of Radiology, The Affiliated Hospital of Qingdao University, Qingdao, Shandong China

## Introduction

IH (internal hernia) is a protrusion of an abdominal viscera from an orifice in the peritoneal cavity.^1^ It is more dangerous than traditional ASBO (adhesive small bowel obstruction) because it is more likely to suffer bowel ischemia^2^ and non-operative treatment failures.^3^ CT (computed tomography) is the most crucial diagnostic tool in IH. Our study aims to build a diagnostic model for IH combined with the clinical parameters and non-enhanced CT findings to improve the diagnostic ability of IH.

## Methods

All patients whose diagnosis of SBO (small bowel obstruction) was confirmed by intraoperative findings from January 1st, 2018, to October 31st, 2021, in *the Affiliated Hospital of Qingdao University* were retrospectively reviewed. Patients who suffered SBO caused by tumor**s**, volvulus, intussusception, or external hernia were excluded. Patients’ information from November 1st, 2021, to July 1st, 2022, were used as prospective validation. Patients’ data in *Qingdao Central Hospital* from January 1st, 2018, to December 31st, 2021, were collected as an external validation cohort. All included patients were divided into IH and ASBO group according to the intraoperative findings.

LASSO (least absolute shrinkage and selection operator) regression was used to select all predictive variables. Our diagnostic model was shown as a nomogram using selected variables. The final model obtained from the derivation groups is applied to the external validation group. R (version 4.0.3) was used for all analyses.

## Results

**Fig. 1:**
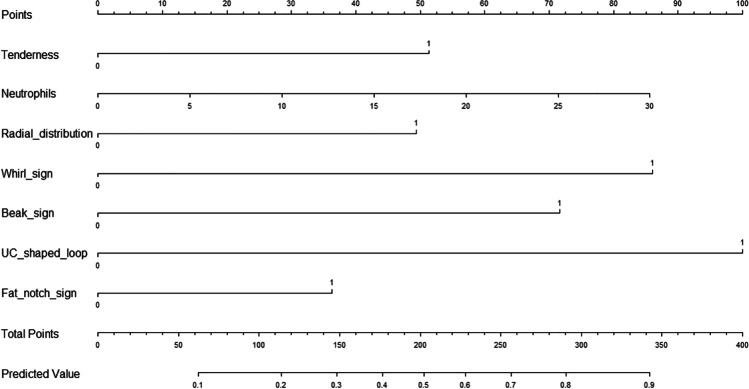
Nomogram to predict the probabilities of IH. Points are assigned by drawing a line upward called the “points” line. The sum of these points, plotted on the “total points” line, corresponds to predictions of IH. If a patient has tenderness and his or her neutrophils were 10*109/L, and abdominal non-enhanced CT scan showed U/C-shaped loop and one beak sign, without radial distribution, whirl sign, and fat notch sign. The total point of the patient was 252, with a predicted value of approximately 68%

Overall, 1008 cases who underwent surgery with the diagnosis of SBO were reviewed. There are 54 and 124 patients diagnosed with IH and ASBO, respectively. Twenty-eight patients (51.8%) in IH group underwent surgeries staying in hospital for less than 1 day, and median duration of patients staying in hospital before operation in ASBO group was 3 days. Thirty-four (66.6%) and 70 (56.5%) patients in the IH and ASBO group underwent small bowel resections. LASSO regression’s λ value was set as 5. Finally, the nomogram (Fig. 1) was built by 7 variables: tenderness, neutrophils, and five CT signs containing radial distribution, whirl sign, beak sign, U/C-shaped loop, and fat notch sign. The Harrell’s C statistic is 0.801(95%*CI* 0.732–0.870). The brier score is 0.160, and *R*2 is 0.317.

Twenty-eight patients were included in our prospective validation, of which 10 were IH confirmed by surgery, and the other 18 patients had ASBO. The Harrell’s C statistic of external validation is 0.903 (95% *CI* 0.7875–1), brier score is 0.175, and *R*2 is 0.360. Sixty-three patients (24IH, 39ASBO) were selected in our external validation. The Harrell’s C statistic of external validation is 0.742 (95% *CI* 0.606–0.877), brier score is 0.195, and *R*2 is 0.203.

## Discussion

**Fig. 2:**
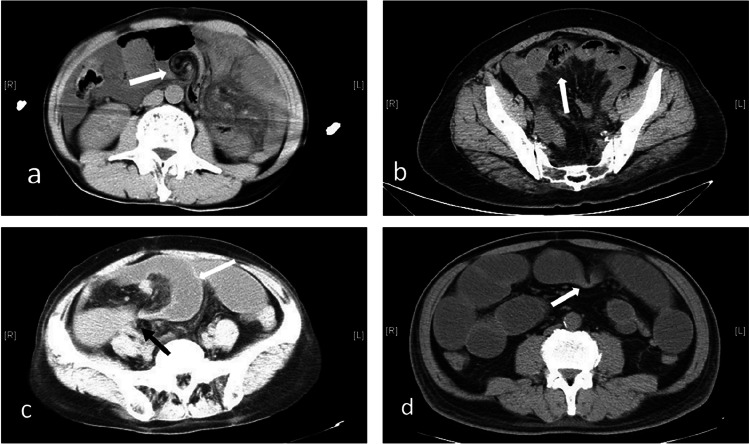
CT signs: **a** whirl sign, **b** radial distribution, **c** U/C-shaped loop (white) and beak sign (black), **d** fat notch sign

Not a single CT sign outperformed the overall impression of the experienced readers in the diagnosis of IH.^4^ So we combine all the CT findings previously reported which account for most of predicted variables in our model (Fig. 2). There are some previous articles focused on the diagnosis of IH. A multicentric retrospective research^5^ built a predictive model for IH after Roux-en-Y Gastric Bypass. But these results cannot apply to other types of IH without the Roux-en-Y anastomosis history. Yen et al.^6^ reported the differences in CT signs between the IH and ASBO group; however, they only described the differences rather than further building a diagnostic model. Moreover, their sample volume is less than ours (78 vs 178).

The limitation of our research is the retrospective study design. The preoperative variables we collected cannot be completely identical to the intraoperative status of the patient. Our model also needs further validation in more patients.
